# Ginsenoside Rb1 targets the NRF2-PPARγ-ACSL4 axis to inhibit PTECs ferroptosis

**DOI:** 10.1186/s13020-025-01292-x

**Published:** 2026-01-08

**Authors:** Binghong Tan, Zhifen Wu, Suwei Wang, Wei Tan, Lirong Lin, Xujia Yang, Luquan Zheng, Jing Li, Ke Li, Jurong Yang, Li Li

**Affiliations:** 1 Department of Nephrology, The Third Affiliated Hospital of Chongqing Medical University, Chongqing, 401120 China; 2 Department of Cardiology, The 924, Hospital of Chinese People’s Liberation Army Joint Service Support Force, Guilin, China; 3 Core Research Laboratory, The Second Affiliated Hospital of Xi’an Jiao Tong University, Xi’an, China; 4 National Key Laboratory for Prevention and Treatment of Multi-Organ Injury, National Clinical Research Center of Kidney Disease, Guangdong Provincial Institute of Nephrology, and Division of Nephrology, Nanfang Hospital, Southern Medical University, Guangzhou, 510515 China

**Keywords:** Ginsenoside Rb1, Ferroptosis, PTEC, NRF2, PPARγ, ACSL4

## Abstract

**Background:**

Ferroptosis, an iron-dependent form of regulated cell death driven by lipid peroxidation, has emerged as a critical pathological mechanism in acute kidney injury (AKI). While pharmacologic targeting of ferroptosis holds therapeutic potential, clinically applicable inhibitors remain elusive, with even the classical inhibitor ferrostatin-1 (Fer-1) demonstrating limitations. Ginsenoside Rb1 (Rb1), a major active component of Panax ginseng, has recently been shown to inhibit ferroptosis in non-renal tissues. This study aimed to investigate the role and mechanism of Rb1 in treating AKI.

**Methods:**

The protective and anti-ferroptotic effects of Rb1 on AKI were evaluated by assessing renal function, tissue damage, inflammation, ferrous iron, glutathione, malondialdehyde, and ferroptosis markers in C57BL/6 mice, as well as cell viability and ferroptosis-related indicators in HK-2 cells. Network pharmacology and molecular docking were employed to identify Rb1's target proteins. Transcriptome sequencing predicted further mechanisms underlying its anti-ferroptotic effects, which were subsequently validated through in vivo and in vitro experiments.

**Results:**

The experimental results demonstrated that Rb1 administration significantly ameliorated renal dysfunction, attenuated tubular necrosis and inflammatory responses, while markedly suppressing ferroptosis-related indicators. Strikingly, Rb1 exhibited superior efficacy to Fer-1 in preventing ferroptosis in proximal tubular epithelial cells (PTECs) in vitro. Nuclear factor erythroid 2-related factor 2 (NRF2) was verified as a direct target for Rb1's ferroptosis-inhibitory effect. Mechanistic studies revealed that Rb1 selectively inhibits lipid peroxidation—the biochemical hallmark of ferroptosis—by activating the NRF2-PPARγ-ACSL4 axis. ﻿

**Conclusion:**

Given its established safety profile in human use, Rb1 represents a potential therapeutic agent for preventing and treating AKI, providing scientific evidence for its application in anti-ferroptosis therapy.﻿

## Introduction

Acute kidney injury (AKI), characterized by rapid deterioration of renal function, affects over 13 million patients annually worldwide [1]. AKI not only imposes substantial healthcare burdens but also markedly elevates risks for chronic kidney disease progression and mortality [2]. Current management remains limited to supportive care, underscoring the urgent need for targeted therapeutics [2].

Mounting evidence implicates multiple regulated cell death pathways—including apoptosis, pyroptosis, necroptosis, and ferroptosis—in AKI pathogenesis [3, 4]. Among these, ferroptosis has emerged as a predominant driver of acute tubular necrosis, distinguished by its iron-dependent accumulation of lethal lipid peroxides [5]. This process is initiated when ferrous ions catalyze reactive oxygen species (ROS) generation via Fenton chemistry, while acyl-CoA synthetase long-chain family member 4 (ACSL4) promotes biosynthesis of peroxidation-susceptible polyunsaturated fatty acid phospholipids (PUFA-PLs) [6, 7]. Compromised antioxidant defenses—particularly through the GPX4-glutathione axis and system xc-cystine/glutamate antiporter (SLC7A11)—further precipitate ferroptotic cell death [8]. Genetic studies confirm that GPX4 deletion or ACSL4 deficiency exacerbates AKI severity [9, 10], establishing ferroptosis as a compelling therapeutic target. However, clinical translation has been hindered by limitations of prototype inhibitors like ferrostatin-1 (Fer-1), including poor pharmacokinetics and incomplete pathway coverage [11].

Ginsenoside Rb1 (Rb1), a principal bioactive component of Panax ginseng, exhibits multimodal therapeutic potential through antioxidative, anti-inflammatory, and metabolic regulatory properties [12]. Recent investigations reveal its ferroptosis-inhibitory capacity in extrarenal tissues: in pulmonary and intestinal models, Rb1 attenuates ferroptosis via heme oxygenase-1 modulation [13], while in hypoxic-ischemic brain injury, it confers neuroprotection through oxidative stress mitigation [14]. Notably, despite ginseng's historical use in renal conditions [15], Rb1's therapeutic potential for AKI and its underlying mechanisms remain insufficiently studied.

In this study, we demonstrate that Rb1 confers renal protection in AKI by specifically targeting ferroptosis through the NRF2-PPARγ-ACSL4 axis. Using integrated in vivo and in vitro models, we establish that Rb1 outperforms classical ferroptosis inhibitor Fer-1 in preserving renal function while effectively suppressing lipid peroxidation. Mechanistically, Rb1-activated NRF2 signaling promotes PPARγ-mediated downregulation of ACSL4, thereby blocking the production of peroxidation-prone phospholipids. These findings not only reveal a novel regulatory node in ferroptosis but also position Rb1 as a clinically translatable therapeutic for AKI.

## Methods

### Single-cell RNA sequence analysis

Single-cell integrated analysis of samples GSM5462519, GSM5462520, GSM5462522, GSM5462529, GSM5462530, and GSM5462531 from the GSE180420 dataset was conducted using the Scanpy analytical toolkit, comprising the following procedures: (1) Raw FASTQ files were processed via CellRanger to generate gene expression matrices; (2) Expression matrices were loaded as AnnData objects using sc.read(), retaining cells expressing at least 200 genes and genes detected in a minimum of 3 cells; (3) Unsupervised clustering was performed via the Louvain algorithm; (4) Cellular death modalities were classified by scoring gene sets of distinct cell death pathways using sc.tl.score_genes, followed by categorization based on computed scores. The single-cell data analysis was conducted by Guangzhou Yance Gene Technology Co., Ltd.

### RNA-sequence analysis

Male C57BL/6J mice were divided into four groups: (i) sham group, (ii) Rb1 group, (iii) IRI group, (iv) Rb1 + IRI group. Mice were euthanized at 48 h after IRI, and then the kidneys were harvested. Total RNA of the kidneys was extracted with TRIzol reagent (Invitrogen), according to the manufacturer’s instructions. RNA purification, reverse transcription, library construction and sequencing (1ug of total RNA) were performed at Shanghai Majorbio Bio-Pharm Biotechnology Co., Ltd (Shanghai, China). The sequencing library was prepared on DNBSEQ-T7 platform (PE150) using DNBSEQ-T7RS Reagent Kit (FCLPE150) version 3.0. The expression level of each transcript was calculated according to the transcripts per million reads (TPM) method. RSEM was used to quantify gene abundances. Differential expression analysis was performed using DESeq2 with DEGs meeting the criteria of |Fold change|≥ 2 and an FDR < 0.05 considered statistically significant. GO functional enrichment and KEGG pathway analysis were performed using GOATools and SciPy (Python), respectively. Adjusted P < 0.05 was considered as a significantly enrichment.

### Antibodies and reagents

Ginsenoside Rb1 (#HY-N0039), RSL3 (#HY-100218A) and Ferrostatin-1 (Fer-1, #HY-100579) were purchased from MedChemExpress (Monmouth Junction, USA). The antibodies to GPX4 (#T56959), SLC7A11 (#T57046), and NRF2 (#T55136) were obtained from Abmart (Shanghai, China). The antibody to PPARγ (#ab178860) was obtained from Abcam (Cambridge, UK). The antibodies to FTH1 (#sc-376594), TFRC (#sc-65882), ACSL4 (#sc-365230) were obtained from Santa Cruz Biotechnology (Dallas, USA). Anti-β-actin (#3700T) was purchased from Cell Signaling Technology (Danvers, USA). Bimake 2 × SYBR Green qPCR master mix (LOW ROX) was obtained from Bimake Biotechnology (Shanghai, China). TRIzol was obtained from Accurate Biotechnology (Changsha, China). Reduced Glutathione Content Assay Kit (#BC1175), Malondialdehyde Content Assay Kit (#BC0025) and Ferrous Ion Content Assay Kit (#BC5415) were purchased from Solarbio (Beijing, China). Reactive Oxygen Species Assay Kit (#S0033S) and Lipid Peroxidation Assay Kit with BODIPY 581/591 C11 (#S0043S) were purchased from Beyotime Institute of Biotechnology (Haimen, China). Cell Counting Kit-8 (#BS350A) was purchased from Biosharp Biotechnology Company (Guangzhou, China).

### Cell culture and treatments

HK-2 cells were purchased from CellCook Biotech (Guangzhou, China). The IACUC protocol number is CC4008. They were cultured in DMEM/F12 supplemented with 10% fetal bovine serum and antibiotics (100 U/ml penicillin G and 100 U/ml streptomycin), and incubated at 37 °C with 5% CO2 and 95% air [16]. After being cultured in the absence or presence of Rb1 (40 µM) [17] or Fer-1 (10 µM) [18] for 2 h, the cells were treated with RSL3 (0.5 µM) [18] for 24 h and then collected or stained for subsequent experiments.

### Cell viability assay

For cell viability assay, cells were seeded into 96-well plates at a density of 0.8 × 10^4^ cells per well. Cell viability was measured using Cell Counting Kit-8 (#BS350A), according to the previous reports [19].

### Mice and renal ischemia–reperfusion model

Male C57BL/6J mice (8–10 w, 20–22 g) were obtained from the Animal Experiment Center of Chongqing Medical University and used in all experimental procedures. The mice were housed in a controlled environment with a 12-h light/dark cycle and provided with ad libitum access to food and water. Twenty-four weight-matched C57BL/6 mice were randomly allocated to four groups (*n* = 6). The groups were as follows: Sham, IRI, Rb1 and Rb1 + IRI. Prior to the establishment of ischemia–reperfusion injury induced acute kidney injury (IRI-induced AKI) model, mice received daily intraperitoneal injections of either Rb1 (40 mg/kg) [20] or saline for three consecutive days. For surgical procedures, mice were anesthetized and placed in a prone position on a temperature-controlled surgical table maintained at 37 °C. Bilateral dorsal incisions were made to expose the kidneys, and renal ischemia was induced by clamping both renal arteries for 30 min using non-traumatic vascular clamps [21]. Sham groups underwent identical surgical procedures without renal artery occlusion. Following 48 h of reperfusion, the mice were sacrificed, and both renal tissue and blood samples were collected for subsequent analysis. All experimental procedures were conducted in strict accordance with the guidelines approved by the “Ethics Review Committee for Animal Experimentation” of Chongqing Medical University.

### Renal function and histology

The levels of serum creatinine and BUN were determined to evaluate renal function. 25% of the mice kidney tissues were kept in paraformaldehyde for 48 h, and then dehydrated and embedded in paraffin. Kidney paraffin Sects. (4 μm) were stained with hematoxylin and eosin (H&E) and periodic acid-Schiff (PAS) to assess renal histopathological changes, using an assay kit (Boster Biotechnology Co., Ltd., China). Kidney Sects. (5 per mouse) were viewed and average histopathological score was presented. The severity of renal histopathology (i.e., tubule thinning, dilatation, loss of proximal brush border, and protein casts) was graded using a 7-point scale method (0, normal kidney; 1, less than 10% necrosis; 2, 10%–25% necrosis; 3, 21–40% necrosis; 4, 41–60% necrosis; 5, 61–75% necrosis and 6, > 75% necrosis), as previously described [21]. The assessment was performed in a blinded fashion by two experienced researchers.

### Real-time quantitative PCR

Total RNA was extracted from both mouse renal cortex tissues and HK-2 cells using TRIzol reagent. Subsequently, cDNA synthesis was performed using the Evo M-MLV Reverse Transcription Kit. Real-time quantitative PCR was carried out using the 2 × SYBR Green qPCR Master Mix (LOW ROX), on a QuantStudio real-time PCR system. Gene expression levels were quantified using the comparative threshold cycle (2-ΔΔCT) method [22]. All primer sequences were designed and synthesized by Tsingke Biotechnology Co., Ltd., with the corresponding sequence information provided in Table 1.

**Table 1:** Primers used in this study

Mouse	
NGAL	AATGTCACCTCCATCCTGGTC
	GCCACTTGCACATTGTAGCTC
KIM-1	TGTTGAGAGTGACAGTGGTCTG
	TGTAGCTGTGGGCCTTGTAG
GPX4	TGTGTAAATGGGGACGATGCC
	GACCATAGCGCTTCACCACG
SLC7A11	ACCATCCCCCTTGCAATCTG
	AGAGGGCAACAAAGATCGGG
ACSL4	CCACACTTATGGCCGCTGTT
	GGGCGTCATAGCCTTTCTTG
TFRC	ATGCCGACAATAACATGAAGGC
	ACACGCTTACAATAGCCCAGG
FTH1	TGAGCCCTTTGCAACTTCGT
	GCGTCCTGGTGGTAGTTCTG
NRF2	CAGCATAGAGCAGGACATGGAG
	GAACAGCGGTAGTATCAGCCAG
PPARγ	TGTTCGCCAAGGTGCTCC AG
	AGGCTCATGTCTGTCTCTGTCTTC
Human	
GPX4	GAGATCAAAGAGTTCGCCGC
	GGAGAGACGGTGTCCAAACT
SLC7A11	ATGCTGGCTGGTTTTACCTCA
	GAAAAGGTCACTGCCACTGC
ACSL4	TTCCTCCAAGTAGACCAACGC
	TCGGTCCCAGTCCAGGTATT
TFRC	GGCTACTTGGGCTATTGTAAAGG
	CAGTTTCTCCGACAACTTTCTCT
FTH1	CCATGTCTTACTACTTTGACC
	GTCTGGTTTCTTGATATCCTG
NRF2	GCCAACTACTCCCAGGTTTCT
	ACCGTCTAAATCAACAGGGGC
PPARγ	CAGAAATGCCTTGCAGTGGG
	ACTCTGGATTCAGCTGGTCG

### Western blotting

For western blot analysis, total protein lysates were extracted from HK-2 cells and renal cortex tissues. Protein samples were separated by SDS-PAGE and transferred onto PVDF membranes using standard protocols. The membranes were then incubated overnight at 4 °C, as described previously [22], with the following primary antibodies: anti-GPX4 (Abmart, #T56959, 1:2000), anti-SLC7A11 (Abmart, #T57046, 1:2000), anti-TFRC (Santa, #sc-65882, 1:1000), anti-FTH1 (Santa, #sc-376594, 1:1000), anti-NRF2 (Abmart, #T55136, 1:1000), anti-PPARγ (Abcam, #ab178860, 1:2000), anti-ACSL4 (Santa, #sc-365230, 1:1000) and anti-β-actin (Cell Signaling Technology, #3700T, 1:1000). After washing, the membranes were incubated with appropriate horseradish peroxidase-conjugated secondary antibodies at room temperature. Protein bands were visualized using an enhanced chemiluminescence (ECL) detection system (GE Healthcare, USA). Band intensity quantification was performed using ImageJ software (National Institutes of Health, Bethesda, MD, USA).

### Malondialdehyde (MDA) and glutathione (GSH) test

According to the manufacturer’s instructions, MDA (#BC0025) and GSH (#BC1175) activity assay kits were used to determine the levels of MDA and GSH in cells and kidney tissues, respectively.

### Reactive oxygen species and lipid ROS detection

Reactive Oxygen Species Assay Kit (#S0033S) and Lipid Peroxidation Assay Kit with BODIPY 581/591 C11 (#S0043S) were used to assess intracellular ROS level and lipid ROS level. According to the reagent manufacturer's instructions, the cells were incubated with 10 μmol/L DCFH-DA fluorescent probe and 2 μM BODIPY 581/591 C11 probe separately for 30 min at 37 ℃ in dark, and washed three times with serum-free medium. Pictures were taken with a fluorescence microscope. Firstly, we use Image J for fluorescence quantitative statistics. Each group uses three images, each of which captures a field of vision of the same area, and then uses GraphPad Prism 9.0 software for data statistics.

### Ferrous ion (Fe^2+^) level analysis

Ferrous Ion Content Assay Kit (#BC5415) was used to detect the ferrous ion content of HK-2 cells and kidney tissues of mice in each group. The specific implementation method was in accordance with the instructions provided by the reagent manufacturer. We use Image J and GraphPad Prism 9.0 software for data statistics.

### Chromatin immunoprecipitation (ChIP) assay

ChIP assay was performed according to the protocol from the SimpleChIP Enzymatic Chromatin IP Kit (Cell Signaling Technology, #9003S). Briefly, cells were fixed for 10 min at room temperature in 1% paraformaldehyde to cross‐link proteins to DNA. The chromatin was harvested using enzymic digestion. An aliquot of each sample was set aside as input control, while the remaining portion was immunoprecipitated with antibodies against NRF2 overnight at 4 °C, with normal rabbit IgG as control. Immune complexes were washed in ChIP buffer, and the DNA‐protein cross‐links was reversed by addition of NaCl and kept at 65 °C for 2 h. After proteinase K digestion, DNA was purified with columns and then amplified by ChIP-qPCR using the following primers: F, 5′- GTTGTCTGAGTCCCTCGGTG −3′; and R, 5′- TCTTCTGATCGGTGGTGCTG −3′.

### Network pharmacology analysis and molecular docking

The possible target genes of Rb1 were retrieved from the SuperPred databases and Swiss Target Prediction databases, and after integration and de-duplication, they were used for the next step of research. The targets related to AKI were retrieved from the GeneCards, DisGeNET, and OMIM databases with "Acute kidney injury" as the keyword, and then integrated and de-duplicated. The targets related to ferroptosis were obtained from the FerrDb database. The overlapping targets of Rb1, AKI and ferroptosis were obtained using a Venn diagram tool. They were input into the STRING database (version 12.0), with a confidence score > 0.4, to obtain the PPI network relationship, and the MCODE plugin in Cytoscape 3.7.1 software was used to screen the modules of the PPI network. The inferred modules were analyzed using default settings (degree cutoff = 2, node score cutoff = 0.2, K-core = 2, maximum depth = 100), and the top-ranked important module was selected for topological analysis and visualization.

The key targets were selected from the topological analysis results, and their 3D structures were downloaded from the PubChem database. The downloaded protein structures were preprocessed using PyMOL 2.6.0. Molecular docking was performed with Rb1 on the AutodockVina 1.1.2 software platform, and the binding energy was calculated. The molecular docking data were visualized using PyMOL.

### Molecular dynamics simulations

Gromacs2022.3 software was used for molecular dynamics simulation. For small molecule preprocessing, AmberTools22 is used to add GAFF force field to small molecules, while Gaussian 16 W is used to hydrogenate small molecules and calculate RESP potential. Potential data will be added to the topology file of molecular dynamics system. The simulation conditions were carried out at static temperature of 300 K and atmospheric pressure (1 Bar). Amber99sb-ildn was used as force field, water molecules were used as solvent (Tip3p water model), and the total charge of the simulation system was neutralized by adding an appropriate number of Na + ions. The simulation system adopts the steepest descent method to minimize the energy, and then carries out the isothermal isovolumic ensemble (NVT) equilibrium and isothermal isobaric ensemble (NPT) equilibrium for 100,000 steps, respectively, with the coupling constant of 0.1 ps and the duration of 100 ps. Finally, the free molecular dynamics simulation was performed. The process consisted of 5,000,000 steps, the step length was 2 fs, and the total duration was 100 ns. After the simulation was completed, the built-in tool of the software was used to analyze the trajectory, and the root-mean-square variance (RMSD), root-mean-square fluctuation (RMSF) and protein rotation radius of each amino acid trajectory were calculated, combined with the free energy (MMGBSA), free energy topography and other data.

### Cellular thermal shift assay (CETSA)

HK-2 cells (1 × 10^7^) were resuspended in PBS containing protease inhibitors. The cells were then lysed by liquid nitrogen through three cycles of freezing and thawing. The resulting cell lysates were centrifuged at 12,000*g* for 15 min at 4 °C to collect the supernatants. The supernatants were divided into two equal parts and treated with either Rb1 (40 μM) or DMSO at 25 °C for 60 min. Subsequently, the samples incubated with Rb1 or DMSO were divided into 6 equal parts and heated at temperatures of 43, 46, 49, 52, 55 and 58 °C for 3 min each. After heating, the samples were centrifuged at 12,000*g* for 10 min at 4 °C, and the resulting supernatants were collected and boiled with loading buffer. The levels of the target proteins were determined using western blotting.

### Statistical analysis

Data values are expressed as the mean ± standard deviation (SD) of n experiments. One-way ANOVA with multiple comparison test was used for comparisons between multiple groups. P < 0.05 was considered significant. Experiments were repeated a minimum of three times. All data were statistically analyzed using GraphPad Prism 9.0 software (GraphPad Software, Inc., San Diego, CA, USA).

## Results

### Ferroptosis is the predominant cell death pathway in renal tubular cells during IRI-induced AKI

**Fig. 1:**
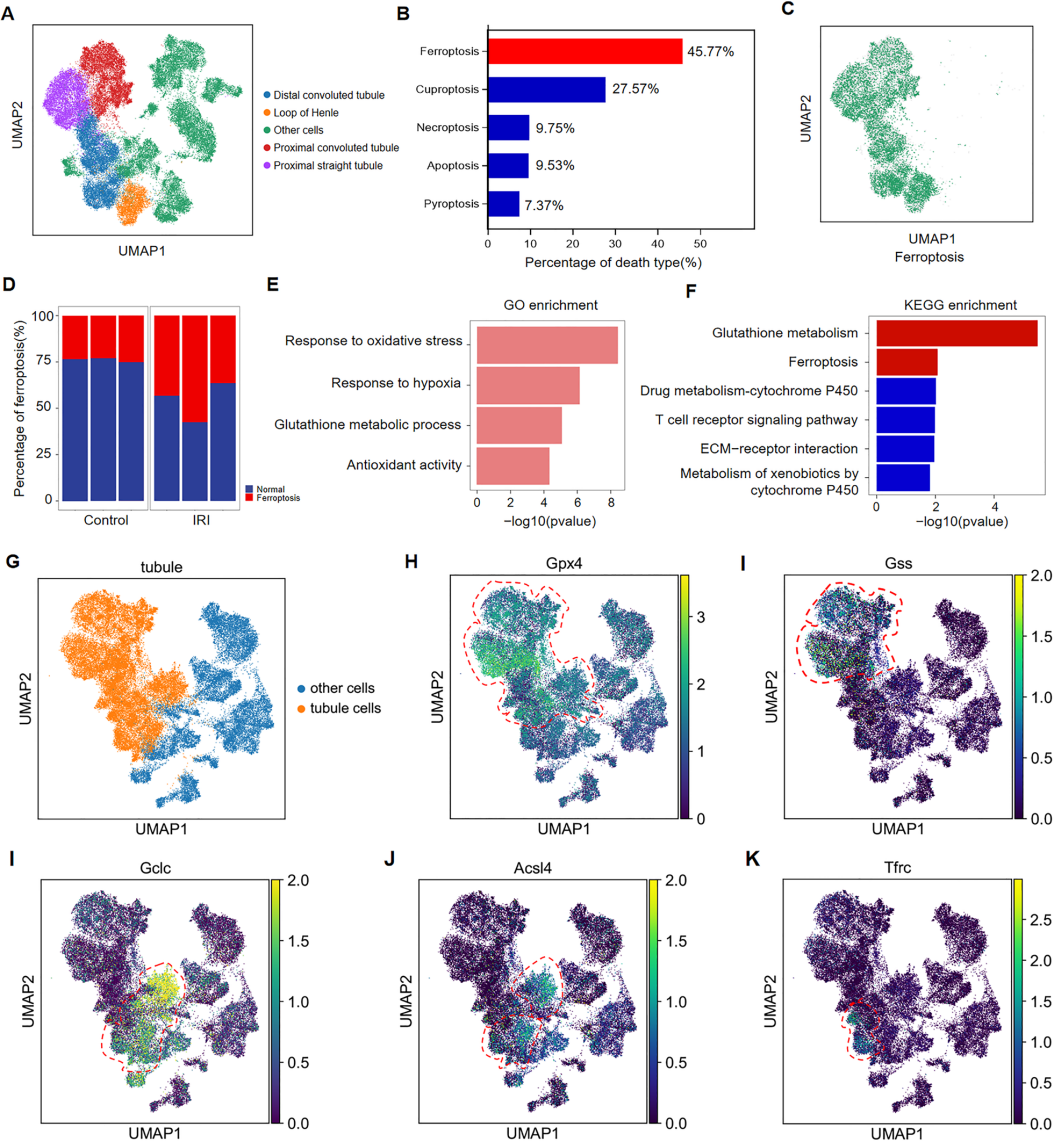
scRNA-seq data revealed ferroptosis is the predominant cell death pathway in renal tubular cells during IRI-induced AKI. **A** UMAP visualization of single cells from the GSE180420 dataset, colored by cluster identity. **B** The proportion of apoptotic, pyroptotic, ferroptotic, cuproptotic and necroptotic tubular cells. **C** UMAP visualization of the spatial distribution of ferroptotic tubular cells. **D** The proportion of ferroptotic tubular cells in different groups. **E–F** GO and KEGG enrichment showing the ferroptosis related pathways. **G**–**K** UMAP visualization of the spatial distribution of GPX4, GSS, GCLC, ACSL4 and TFRC in kidney tissue

Given the well-documented heterogeneity of cell death modalities in AKI [4], we systematically investigated the predominant form as potential therapeutic target by comprehensively characterizing the prevalence and spatial distribution of distinct regulated cell death pathways during AKI pathogenesis. We leveraged publicly available single-cell RNA sequencing (scRNA-seq) data from the GSE180420 dataset, that provides high-resolution transcriptomic profiling across > 110,000 cells throughout AKI progression. Through meticulous cell type annotation using established marker gene expression signatures, we identified and quantified the major renal cellular compartments, including distinct populations of distal convoluted tubule cells, loop of Henle cells, proximal convoluted tubule cells, and proximal straight tubule cells (Fig. 1A). Quantitative deconvolution of cell death patterns revealed ferroptosis as the predominant modality, representing 45.77% of all regulated cell death events (Fig. 1B). Notably, ferroptotic cell death exhibited specific enrichment within renal tubular epithelial cell populations (Fig. 1C), with significantly increased proportions observed following ischemic injury compared to sham controls (Fig. 1D). Functional enrichment analyses provided mechanistic insights into this observation. Gene Ontology (GO) analysis demonstrated significant upregulation of ferroptosis-associated biological processes post-IRI, particularly those related to oxidative stress response, hypoxia adaptation, glutathione metabolism, and antioxidant defense (Fig. 1E). Complementary KEGG pathway analysis further confirmed the specific enrichment of ferroptosis-related pathways, with glutathione metabolism emerging as the most significantly altered metabolic cascade (Fig. 1F). Spatial mapping of key ferroptosis regulatory components, including GPX4, GSS, GCLC, ACSL4 and TFRC revealed their predominant expression within tubular epithelial cell populations (Fig. 1G–K), mirroring the spatial distribution patterns observed for ferroptotic cells. This convergence of bioinformatic evidence strongly supports ferroptosis as the dominant regulated cell death mechanism in IRI-induced AKI pathogenesis. Despite these mechanistic insights, current clinical interventions targeting ferroptosis in AKI remain critically limited. Our findings therefore highlight an urgent need for the development of novel therapeutic agents capable of effectively modulating this cell death pathway in renal injury contexts.

### ﻿Therapeutic efficacy of Rb1 in IRI-induced AKI

**Fig. 2:**
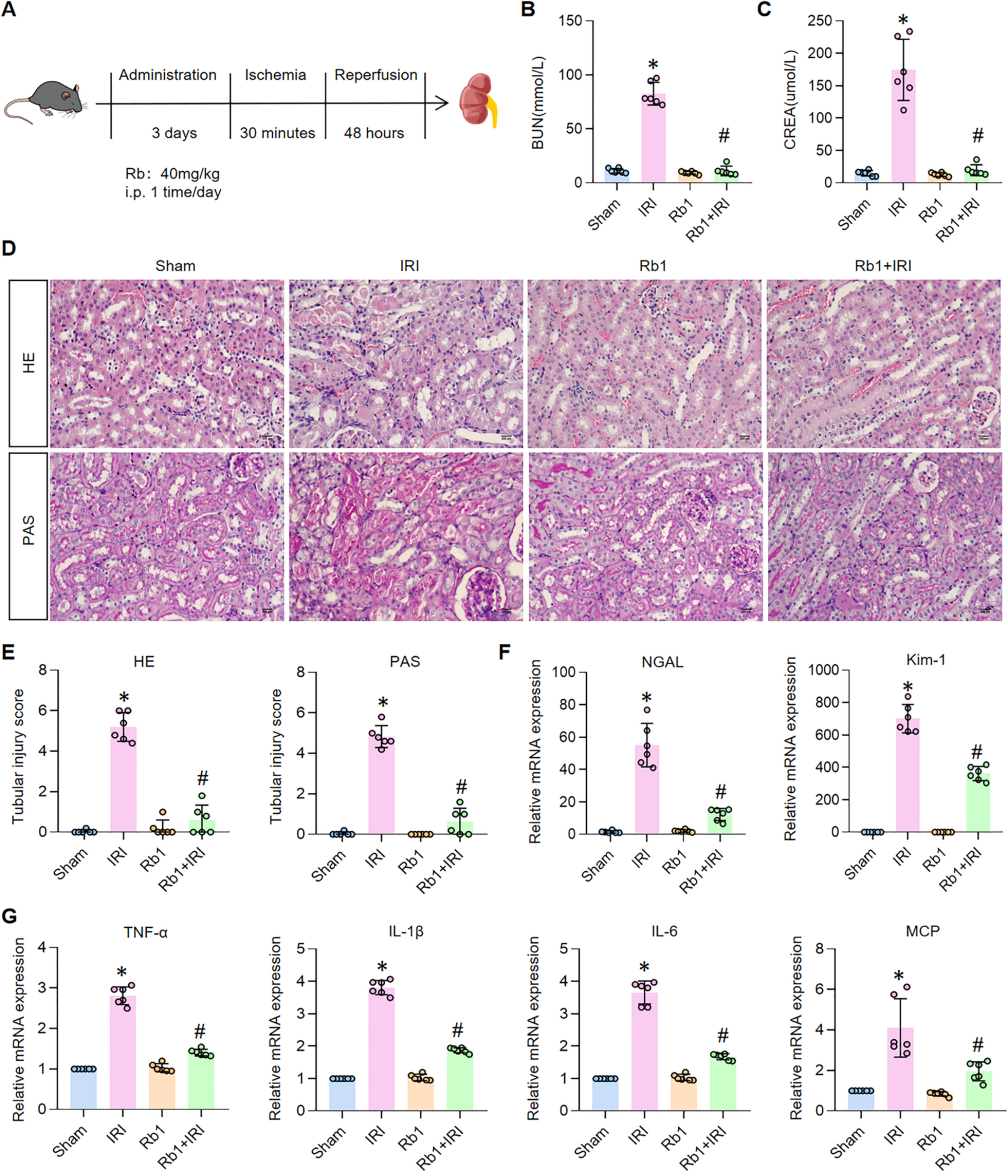
Rb1 attenuated the progression of AKI and improves the inflammatory response in IRI-induced AKI mice. Animals were divided into: Sham group, Rb1 group, IRI group and Rb1 + IRI group. **A** The scheme of IRI-induced AKI model and Rb1 treatment. **B**, **C** BUN and blood CREA levels in mice. n = 6. **D** Representative images for H&E and PAS staining in kidneys (magnification ×400, scale bar 200 μm). **E** Tubular injury scores of H&E and PAS staining. n = 6. **F** Relative mRNA expression of NGAL and Kim-1. n = 6. **G** Relative mRNA expression of TNF-α, IL-1β, IL-6 and MCP. n = 6. *P < 0.05, compared with Sham group; #P < 0.05, compared with IRI group

Given previous reports of ginsenoside Rb1 as a ferroptosis inhibitor with antioxidant and anti-inflammatory properties [13, 14, 23, 24], coupled with our findings establishing ferroptosis as a critical mechanism in IRI-induced AKI (Fig. 1), we investigated whether Rb1 could mitigate kidney injury through ferroptosis inhibition. Using an established IRI-induced AKI model (Fig. 2A) that reflects the most prevalent clinical cause of AKI [25], we first examined the effects of Rb1 in sham-operated mice and found no significant alterations in blood urea nitrogen (BUN) or serum creatinine (CREA) levels (Fig. 2B, C), microscopic observation of H&E and PAS stained sections showed no significant changes in tissue structure (Fig. 2D, E), indicating a favorable safety profile. In contrast, Rb1 treatment in IRI-induced AKI mice resulted in significant improvement in renal function, evidenced by marked reductions in BUN and CREA (Fig. 2B, C), along with substantial histological preservation, including decreased brush border loss, attenuated tubular dilation, diminished protein cast formation, and reduced necrotic changes (Fig. 2D, E). Further supporting its nephroprotective effects, Rb1 administration significantly suppressed the injury-induced upregulation of tubular damage markers Kim-1 and NGAL (Fig. 2F) and reduced mRNA levels of proinflammatory cytokines IL-6, IL-1β, TNF-α, and MCP-1 (Fig. 2G). Together, these data demonstrate that Rb1 effectively attenuates renal dysfunction, tubular injury, and inflammation in IRI-induced AKI, highlighting its potential as a ferroptosis-targeted therapeutic agent.

### Rb1 inhibits ferroptosis in IRI-induced AKI

**Fig. 3:**
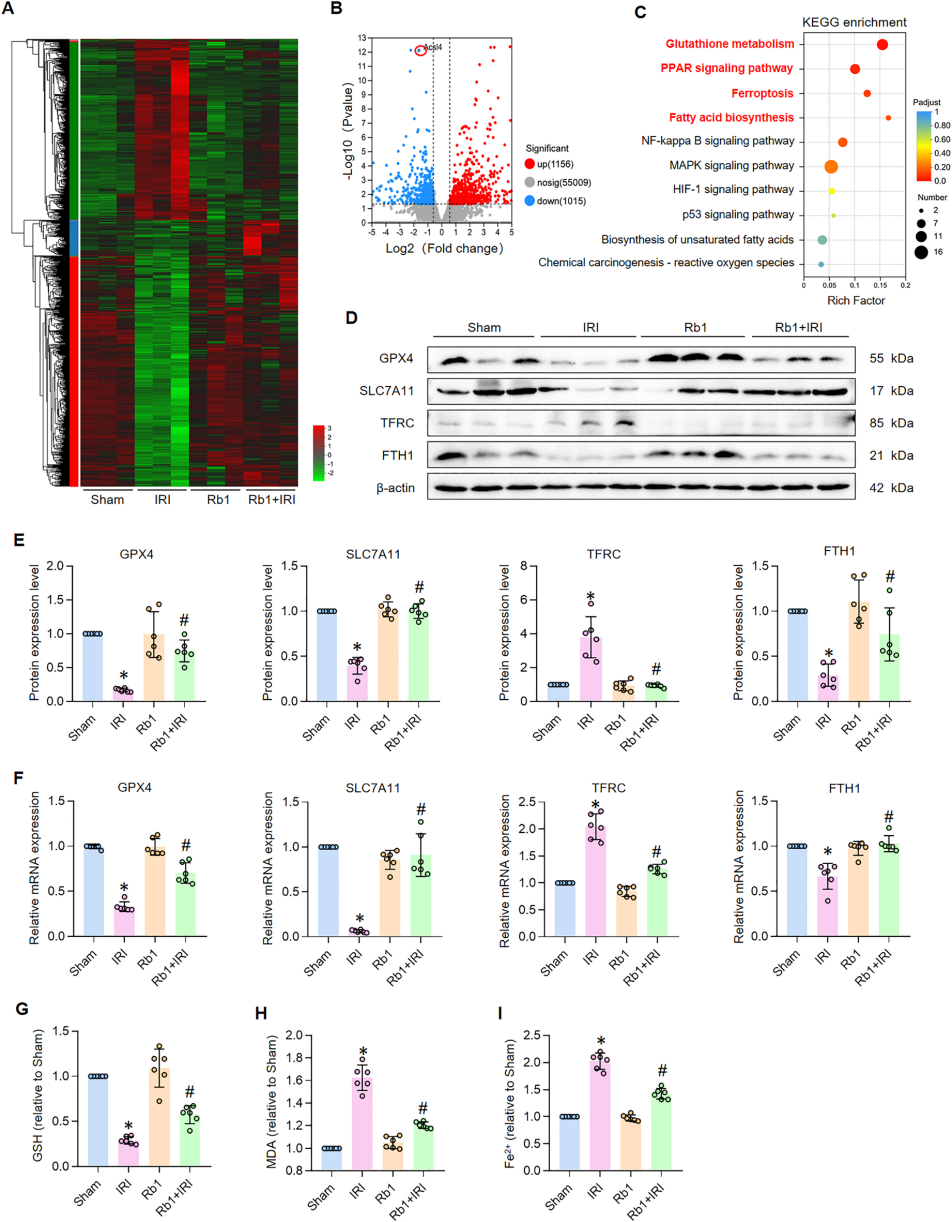
Rb1 inhibits ferroptosis in IRI-induced AKI mice. **A** Heatmap showing DEGs in the different groups assayed by RNA-seq. **B** Volcano plot displaying DEGs in Rb1 + IRI group compared with IRI group. **C** Enrichment analysis of KEGG signaling enriched pathways in Rb1 + IRI group compared with IRI group. **D**, **E** Western bolt results and quantitative analysis of GPX4, SLC7A11, TFRC and FTH1. n = 6. **F** Relative mRNA expression of GPX4, SLC7A11, TFRC and FTH1. n = 6. **G**–**I** GSH, MDA and ferrous iron levels of kidney tissue. n = 6. *P < 0.05, compared with Sham group; #P < 0.05, compared with IRI group

Emerging evidence has demonstrated the ferroptosis-inhibitory effects of ginsenoside Rb1 in pulmonary and neurological contexts [20, 21]. Building upon these findings, we sought to investigate whether Rb1 similarly attenuates ferroptosis in renal tubules following ischemia–reperfusion injury. To address this, we performed transcriptomic profiling via RNA sequencing (RNA-seq) across four experimental groups (Fig. 3A). Differential gene expression (DEG) analysis between the Rb1 + IRI and IRI groups identified 1156 upregulated and 1015 downregulated genes, suggesting a broad regulatory role of Rb1 in renal repair (Fig. 3B). Key insights emerged from KEGG pathway enrichment analysis, which revealed significant associations between Rb1-modulated DEGs and ferroptosis, PPAR signaling, fatty acid biosynthesis, and glutathione metabolism (Fig. 3C). These results strongly indicated that Rb1-mediated renal protection might primarily stem from ferroptosis suppression. For mechanistic validation, we assessed key ferroptosis-associated proteins. Western blot analysis confirmed that Rb1 treatment upregulated the antioxidant regulators GPX4 and SLC7A11, enhanced FTH1 (a ferritin subunit that sequesters free iron), while downregulating the iron importer TFRC (Fig. 3D, E). Concurrently, mRNA expression analysis corroborated these findings (Fig. 3F). Given the pivotal roles of lipid peroxidation and iron dysregulation in ferroptosis, we further quantified relevant metabolites and found Rb1 significantly reduced renal MDA (a marker of oxidative stress) while elevating GSH levels (Fig. 3G, H). Notably, Rb1 also mitigated pathological iron accumulation, as evidenced by decreased ferrous iron deposition in kidney tissues (Fig. 3I). Collectively, these data provide compelling evidence that Rb1 attenuates IRI-induced AKI by inhibiting ferroptosis, likely via dual modulation of oxidative defense and iron metabolism.

### Rb1 exhibits superior ferroptosis inhibition in HK-2 cells compared to Fer-1

**Fig. 4:**
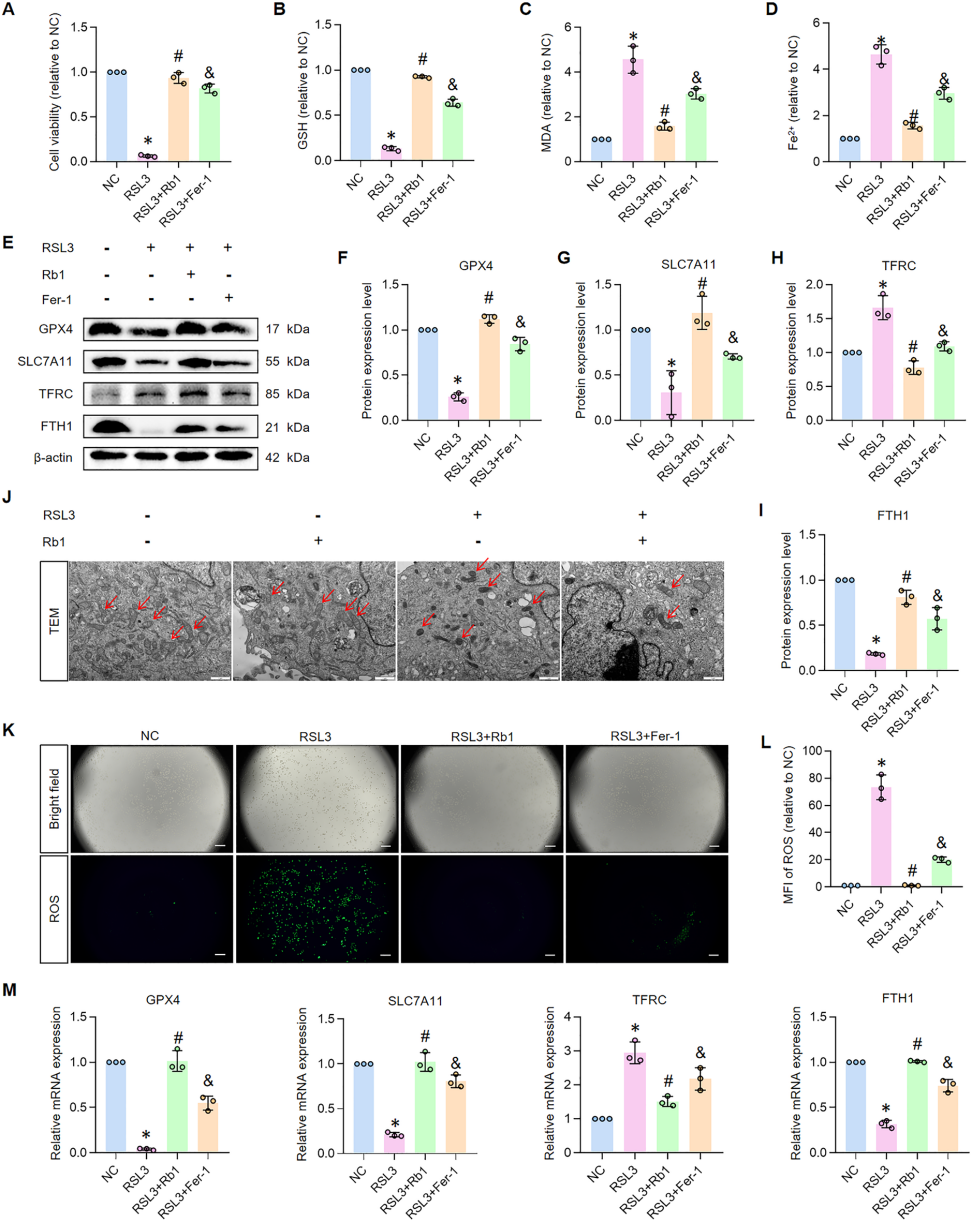
Rb1 inhibits ferroptosis in RSL3-induced HK-2 cells. **A** Cell viability assayed by CCK-8. n = 3. **B**–**D** Ferrous iron, GSH, and MDA levels of HK-2 cells. n = 3. **E**–**I** Western bolt results and quantitative analysis of GPX4, SLC7A11, TFRC and FTH1. n = 3. **J** Representative transmission electron microscopy pictures of HK-2 cells and quantification of relative mitochondrial length (scale bar 1 μm). n = 3. **K**, **L** Representative photomicrographs and quantitative analysis of HK-2 cells stained with DCFH-DA fluorescent probe (magnification ×40, scale bar 200 μm). n = 3. **M** Relative mRNA expression of GPX4, SLC7A11, TFRC and FTH1. n = 3. *P < 0.05, compared with NC group; #P < 0.05, compared with RSL3 group; &P < 0.05, compared with RSL3 + Rb1 group

Given the compelling in vivo evidence for Rb1-mediated ferroptosis suppression, we sought to validate these findings in vitro using human proximal tubular epithelial (HK-2) cells. Ferroptosis was chemically induced by RSL3 treatment, with Fer-1 serving as the reference inhibitor due to its established role in ferroptosis prevention [11]. Remarkably, Rb1 pretreatment not only mitigated RSL3-induced cytotoxicity but demonstrated superior protective efficacy compared to Fer-1 (Fig. 4A), indicating potent anti-ferroptotic activity. Mechanistic investigation revealed that Rb1 treatment significantly attenuated hallmark features of ferroptosis. This was evidenced by substantial reductions in malondialdehyde (MDA) and ferrous iron (Fe^2^⁺) accumulation (Fig. 4C, D), coupled with a concomitant elevation in cellular GSH levels (Fig. 4B)—effects that surpassed those achieved with Fer-1. At the molecular level, immunoblot analyses demonstrated Rb1-mediated upregulation of GPX4, SLC7A11, and FTH1, alongside downregulation of TFRC (Fig. 4E–I). These protein expression patterns were corroborated at the transcriptional level by quantitative PCR (Fig. 4M). Ultrastructural analysis by transmission electron microscopy (TEM) confirmed these observations, with Rb1 pretreatment effectively preventing the characteristic mitochondrial pathology (cristae disruption and organelle shrinkage) induced by RSL3 (Fig. 4J). Furthermore, flow cytometry revealed that Rb1 more effectively suppressed RSL3-induced ROS accumulation compared to Fer-1 (Fig. 4K, L), further supporting its superior antioxidant capacity.

These comprehensive in vitro findings reinforce and extend our in vivo observations, demonstrating that Rb1 exerts multifaceted protection against PTECs ferroptosis through coordinated modulation of lipid peroxidation, iron homeostasis, and redox balance. The consistent superiority of Rb1 over the canonical ferroptosis inhibitor Fer-1 positions this natural compound as a particularly promising therapeutic candidate for acute kidney injury intervention.

### Identification of key genes mediating the anti-ferroptotic effects of Rb1

**Fig. 5:**
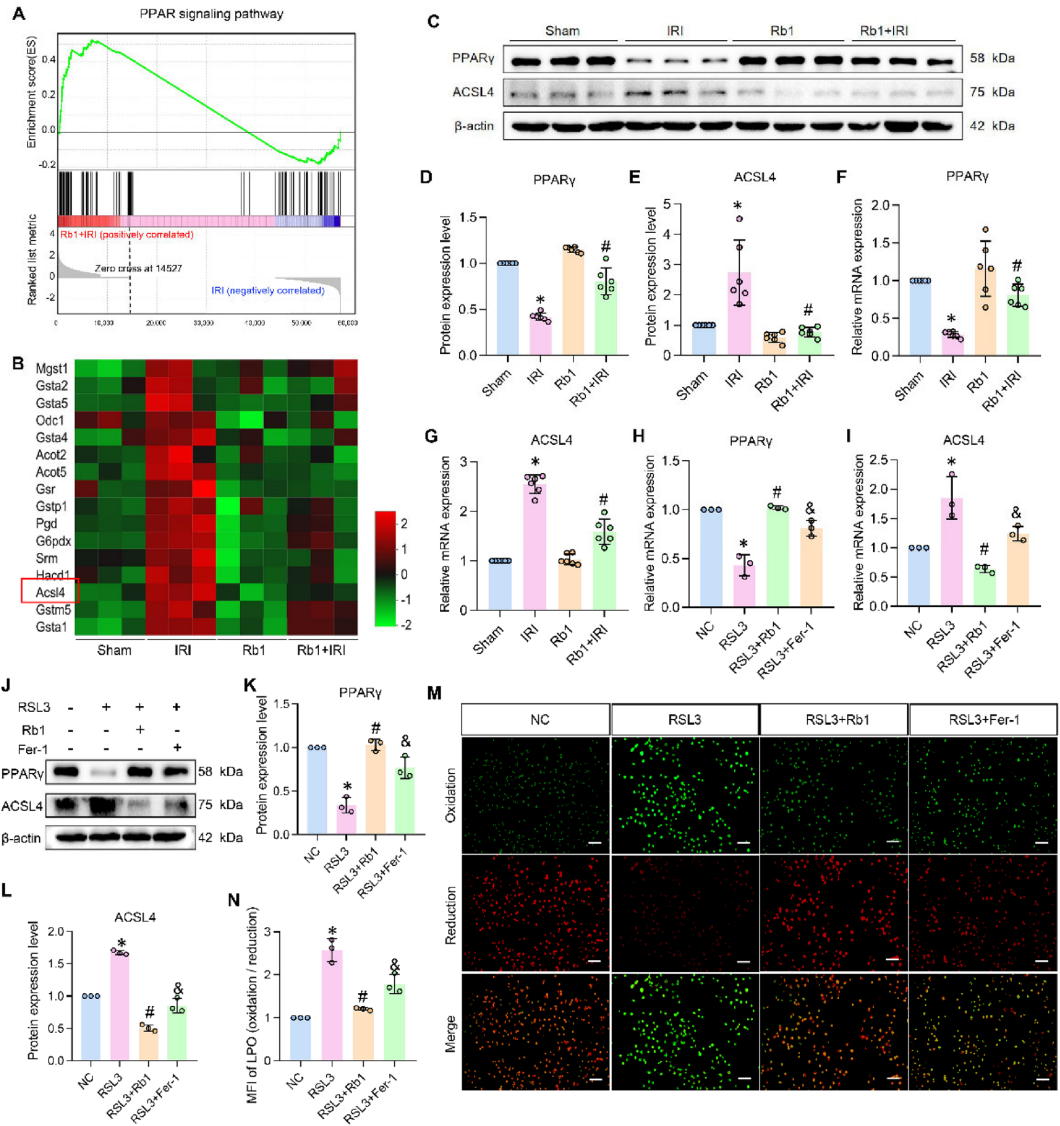
PPARγ and ACSL4 were the core gene in Rb1-mediated ferroptosis inhibition. **A** GSEA showing the increased PPAR signaling pathway enrichment in Rb1 + IRI group compared with IRI group. **B** Heatmap displaying ferroptosis related DEGs from RNA-seq data. **C**–**E** Western bolt results and quantitative analysis of PPARγ and ACSL4 in vivo. n = 6. **F**–**I** Relative mRNA expression of PPARγ and ACSL4 in vivo and vitro. **J**–**L** Western bolt results and quantitative analysis of PPARγ and ACSL4 in vitro. n = 3. **N**, **M** Representative photomicrographs and quantitative analysis of HK-2 cells stained with C11-Bodipy fluorescent probe (magnification ×100, scale bar 200 μm). n = 3. *P < 0.05, compared with Sham group or with NC group; #P < 0.05, compared with IRI group or with RSL3 group; &P < 0.05, compared with RSL3 + Rb1 group

**Fig. 6:**
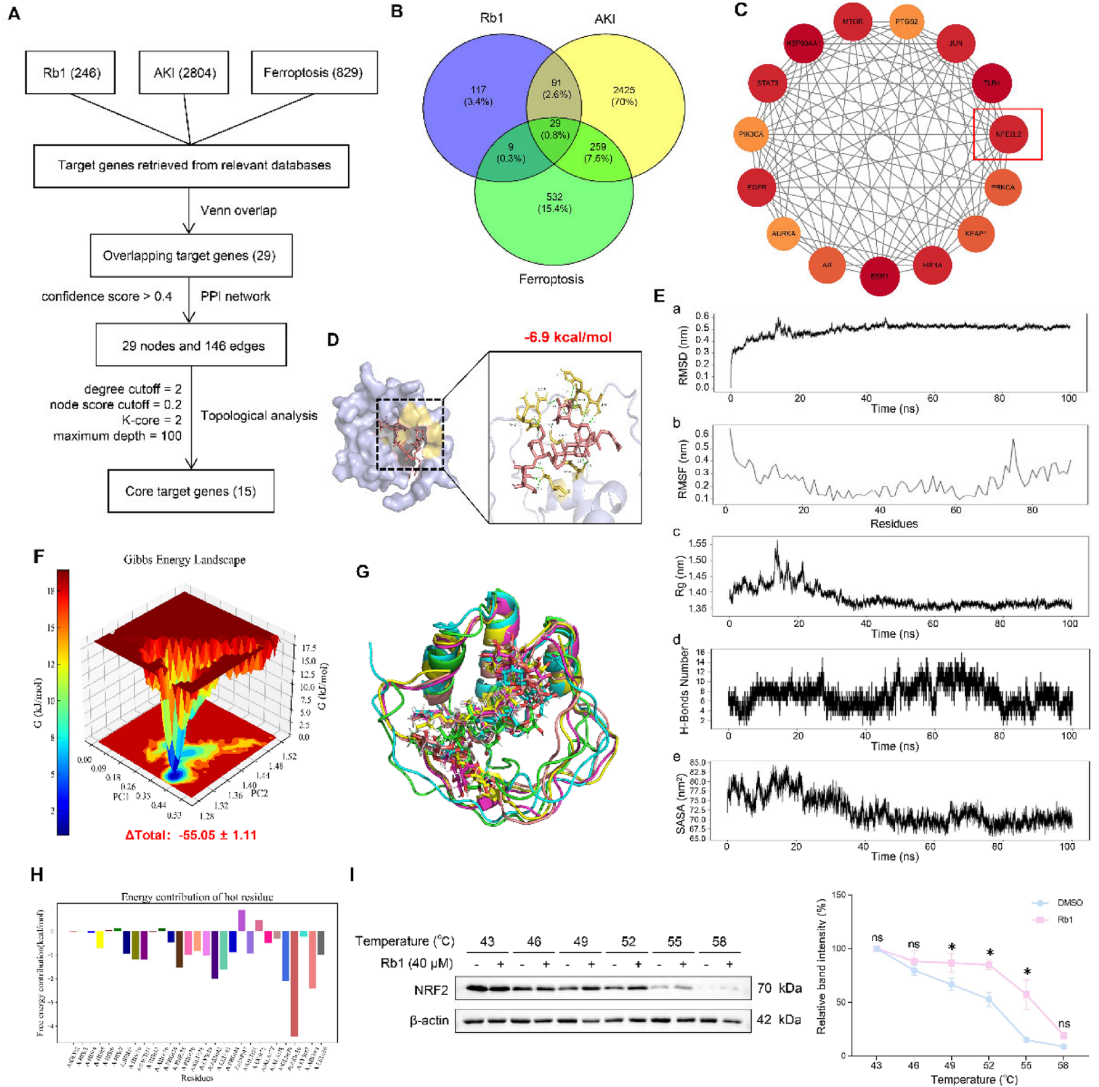
NRF2 was the key transcription factor in Rb1-mediated ferroptosis inhibition. **A** Flow chart showing the process of network pharmacology analysis. **B** Venn diagram illustrating the overlapped targets identified among Rb1, AKI, and ferroptosis. **C** PPI network displaying the top 15 core targets. **D** Molecular docking and docking scores of Rb1 to the catalytic core of NRF2. **E** (a) Root mean square deviation (RMSD), (b) root mean square fluctuation (RMSF), (c) Radius of gyration (Rg), (d) Hydrogen bond counts, and (e) solvent-accessible surface area (SASA) curves of the NRF2-Rb1 complex. **F** Free energy landscape of the NRF2-Rb1 complex. **G** Structural comparison of the NRF2-Rb1 complex at six time points (0, 20, 40, 60, 80, 100 ns) during molecular dynamics simulations. **H** Per-residue energy decomposition analysis of NRF2 residues involved in Rb1 binding. **I** CETSA-Western blot analysis showed the protection of NRF2 by Rb1 at different temperature gradients (n = 3). *P < 0.05, compared with NC group

After establishing the crucial role of ferroptosis in Rb1-mediated renal tubular epithelial cell protection, we aimed to identify key genes involved in the ferroptosis process. The KEGG enrichment analysis revealed that Rb1-modulated genes were significantly associated with the PPAR signaling pathway and unsaturated fatty acid biosynthesis (Fig. 3C). Given that PPARγ, as a key subtype of peroxisome proliferator-activated receptors (PPARs), plays a crucial regulatory role in unsaturated fatty acid metabolism [26, 27], we systematically investigated whether Rb1's ferroptosis inhibition mechanism involves PPARγ-mediated lipid metabolism regulation and identified potential direct molecular targets. Firstly, Parallel analysis of our RNA-seq data through gene set enrichment analysis (GSEA) confirmed significant activation of PPAR signaling pathway in Rb1-treated groups (Fig. 5A), reinforcing the KEGG findings. Since ACSL4-mediated biosynthesis of polyunsaturated fatty acids (PL-PUFAs), particularly the long-chain oxidation-prone species, represents a critical determinant of ferroptotic susceptibility [28, 29], we specifically examined its regulation by the PPARγ pathway. Comparative transcriptomic analysis demonstrated that ACSL4 expression was markedly reduced in Rb1-treated groups compared to IRI controls (Fig. 5B), a finding that was rigorously validated through Western blot and quantitative PCR analyses, which collectively showed that Rb1 treatment significantly upregulated PPARγ while simultaneously downregulating ACSL4 expression in vitro and in vivo experiments (Fig. 5C–L). Given ACSL4's established function in catalyzing the biosynthesis of oxidation-prone polyunsaturated fatty acids [29], we further evaluated Rb1's impact on lipid peroxidation using C11-BODIPY fluorescence assays. Quantitative analysis demonstrated that Rb1 treatment significantly suppressed RSL3-induced lipid ROS accumulation (Fig. 6N, M), outperforming Fer-1 in preventing peroxidative damage. These findings highlight PPARγ and ACSL4 as key ferroptosis-related genes, implicating them as promising therapeutic targets for alleviating IRI-induced AKI.

### NRF2 as a key regulator modulating PPARγ in Rb1-conferred anti-ferroptosis

To identify the binding target proteins of Rb1 and to determine the potential transcription factors (TFs) regulating the differentially expressed genes (DEGs) in AKI and ferroptosis, we performed virtual screening and network pharmacology analysis. Firstly, Venn diagram analysis identified 29 overlapping targets from Rb1-related targets (246), AKI-associated genes (2804), and ferroptosis-related genes (829) (Fig. 6B). Subsequent protein–protein interaction (PPI) network construction using the STRING database revealed complex interactions among these targets (29 nodes with 146 edges) (Fig. 6C). Through network pharmacology analysis and topology analysis using Cytoscape software, 15 core targets were identified, including HSP90AA1, ESR1, TLR4, NFE2L2 (NRF2), STAT3, and MTOR (Fig. 6A, Table 2). Molecular docking studies demonstrated that among these candidates, NRF2 exhibited the strongest binding affinity with Rb1 (minimum binding energy:  − 6.9 kcal/mol, with values < − 5.0 kcal/mol indicating significant binding activity) (Fig. 6D).

**Table 2:** MCODE scores of core targets identified by topological analysis

MCODE score	Gene name
9.49	HSP90AA1
9.49	ESR1
9.49	TLR4
9.23	NFE2L2
9.23	STAT3
9.23	EGFR
9.23	HIF1A
9.23	MTOR
9.23	JUN
8.84	AR
8.84	PRKCA
8.84	KEAP1
8.31	PIK3CA
8.31	PTGS2
8.00	AURKA

Subsequently, we performed molecular dynamics simulations to further validate the binding stability between NRF2 and Rb1, which revealed a high degree of stability. The complex exhibited an average RMSD of 0.50 nm ± 0.05 nm, fluctuating within a 1 nm range, indicating structural stability (Fig. 6E-a). The average RMSF for NRF2 was 0.23 nm ± 0.11 nm, suggesting limited residue fluctuation. particularly as the key binding regions displayed moderate flexibility, which is conducive to maintaining the structural integrity of the active site (Fig. 6E-b). The average Rg value of the NRF2-Rb1 complex was 1.38 nm ± 0.03 nm, demonstrating that the complex maintained a compact conformational state with minimal fluctuation, reflecting excellent overall structural stability (Fig. 6E-c). Furthermore, an average of 7 ± 2 hydrogen bonds were formed between NRF2 and Rb1. This stable hydrogen-bond network provided a solid foundation for the interaction and enhanced the anchoring of the ligand (Fig. 6E-d). The average Solvent Accessible Surface Area (SASA) was 72.42 nm^2^ ± 3.80 nm^2^, further corroborating the stability of the system (Fig. 6E-e). The free energy landscape of the NRF2-Rb1 complex was characterized by a single, relatively concentrated global minimum energy basin, indicative of a stable complex (Fig. 6F). Trajectory analysis at 0, 20, 40, 60, 80, and 100 ns confirmed that Rb1 remained bound to NRF2 at the same site without significant positional shifts, demonstrating excellent binding stability (Fig. 6G). The calculated total binding free energy was − 55.05 kcal/mol ± 1.11 kcal/mol, suggesting a strong binding affinity between NRF2 and ginsenoside Rb1 (Fig. 6F). Rb1 exhibited favorable binding with NRF2 residues Asn0, Arg84, Gln79 and Asn42, with binding energies of − 4.435 kcal/mol, − 2.403 kcal/mol, − 2.11 kcal/mol and − 2.002 kcal/mol, respectively, highlighting their key roles in the interaction (Fig. 6H). Consistently, CETSA analysis also evidenced that Rb1 markedly protected NRF2 protein from temperature-dependent denaturation (Fig. 6I). Collectively, these findings establish the structural stability, dynamic equilibrium, and high binding affinity of the NRF2-Rb1 complex.

**Fig. 7:**
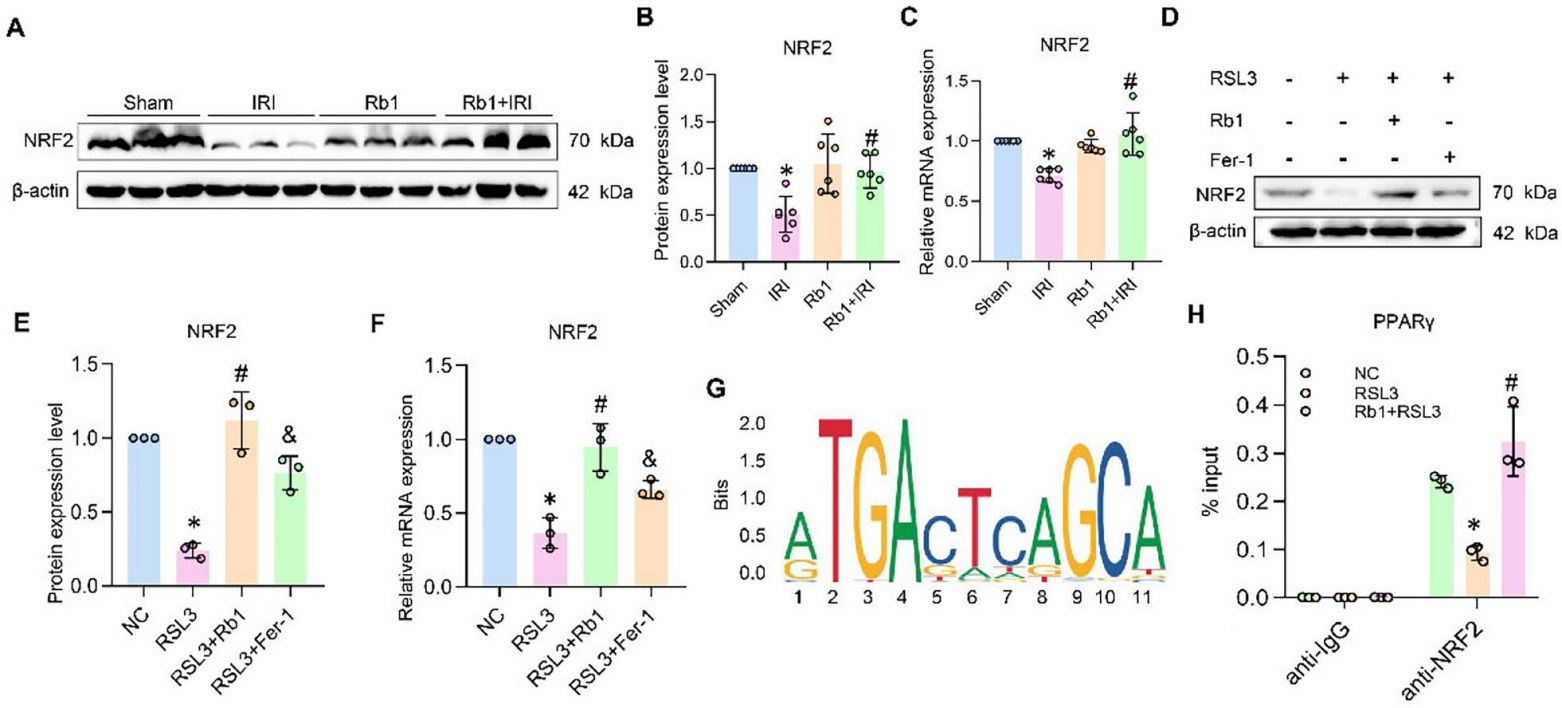
NRF2 as a key regulator modulating PPARγ in Rb1-conferred anti-ferroptosis. **A**–**F** Western bolt results, its quantitative analysis and relative mRNA expression of NRF2 in vivo and vitro. **G** Binding motifs of NRF2 from the JASPAR database. **H** ChIP assay analysis of NRF2 binding to PPARγ in HK-2 cells treated with or without RSL3 and Rb1. *P < 0.05, compared with Sham group or with NC group; #P < 0.05, compared with IRI group or with RSL3 group; &P < 0.05, compared with RSL3 + Rb1 group

To experimentally validate these findings, we performed Western blot and RT-qPCR analyses. Results showed that Rb1 treatment significantly upregulated both the protein and mRNA expression levels of NRF2 compared to the IRI control group (Fig. 7A–F). Notably, in vitro experiments, the effect of Rb1 was superior to that of Fer-1. We further analyzed NRF2 information from the JASPAR database and visualized the conserved binding motif (Fig. 7G). ChIP assays confirmed that NRF2 regulated PPARγ expression by binding to its promoter. In the NC group, immunoprecipitation with an anti-NRF2 antibody enriched abundant PPARγ promoter DNA fragments. This enrichment was significantly reduced in the RSL3-induced ferroptosis model in HK-2 cells, however, pretreatment with Rb1 reversed this reduction, indicating that Rb1 promoted the binding of NRF2 to the PPARγ promoter, leading to the upregulation of PPARγ expression (Fig. 7H). In summary, these findings demonstrate that Rb1 confers anti-ferroptotic and renoprotective effects on tubular epithelial cells by directly binding to NRF2, upregulating its expression, and activating the NRF2-PPARγ pathway.

### Rb1-mediated ferroptosis inhibition through NRF2-PPARγ-ACSL4 axis

**Fig. 8:**
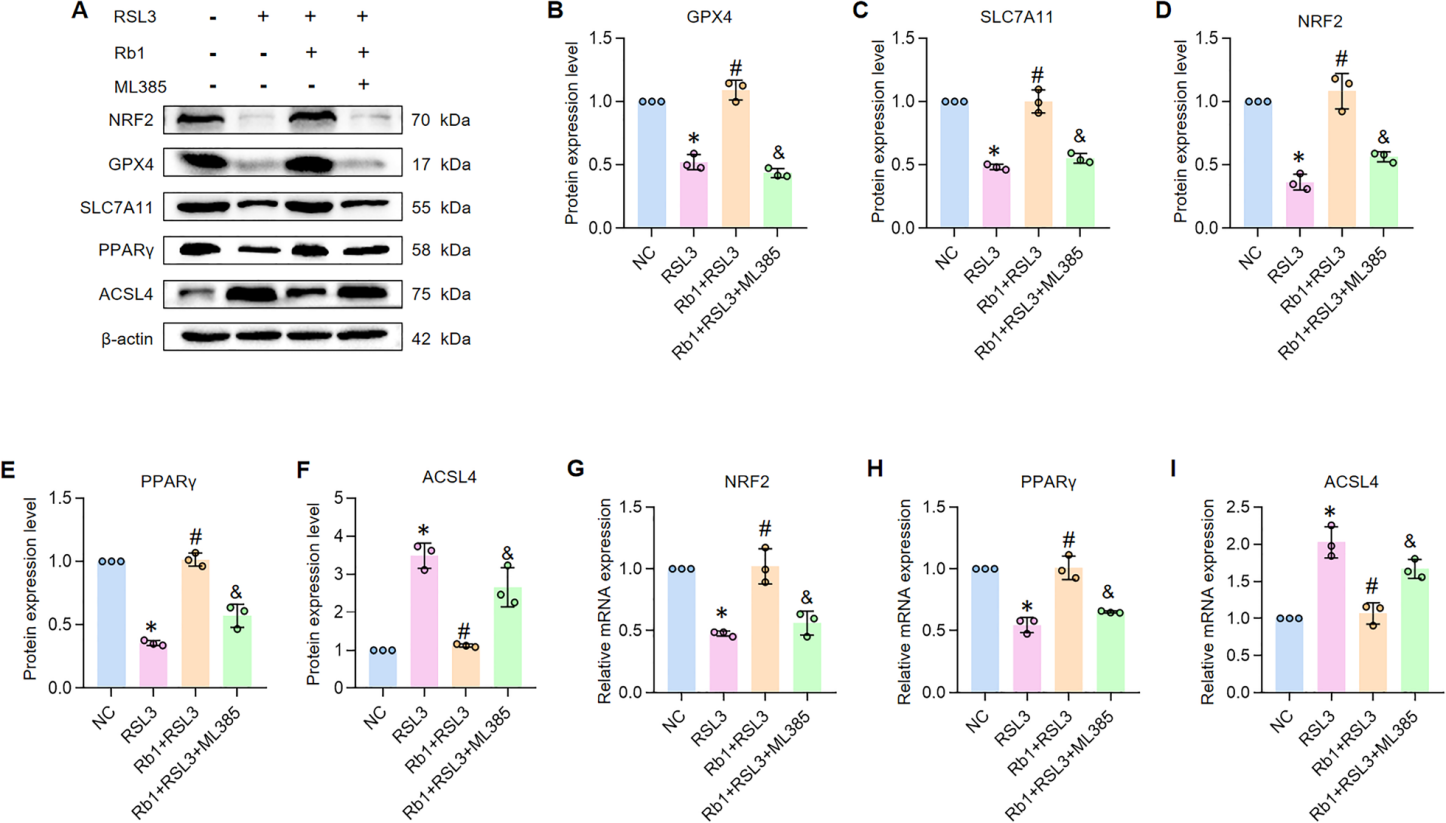
ML385 abolished the NRF2-PPARγ-ACSL4 axis and the anti-ferroptosis effect of Rb1. **A**–**F** Western bolt results and quantitative analysis of GPX4, SLC7A11, NRF2, PPARγ and ACSL4 in vitro. n = 3. **G**–**I** Relative mRNA expression of NRF2, PPARγ and ACSL4 in vitro. n = 3. *P < 0.05, compared with NC group; #P < 0.05, compared with RSL3 group; &P < 0.05, compared with RSL3 + Rb1 group

To further validate that Rb1 suppresses ferroptosis by activating the NRF2-PPARγ-ACSL4 signaling axis, we pretreated HK-2 cells with ML385 (5 nM), a specific NRF2 inhibitor, 30 min prior to Rb1 administration [30]. The experiment included four groups (n = 3): NC, RSL3, Rb1 + RSL3, and ML385 + Rb1 + RSL3. Western blot and RT-qPCR analyses showed that ML385 suppressed the Rb1-induced upregulation of key anti-ferroptotic markers (GPX4, SLC7A11). Similarly, the upregulation of NRF2 and PPARγ and the downregulation of ACSL4 by Rb1 were largely abolished by ML385 (Fig. 8A–I). Collectively, these findings substantiate that Rb1 confers renal tubular protection by activating the NRF2-PPARγ-ACSL4 axis to effectively block ferroptosis in tubular epithelial cells.

## Discussion

In this study, we validated ferroptosis is a critical pathogenic mechanism in renal tubular epithelial cells during IRI-induced AKI. Through comprehensive in vivo and in vitro investigations, we demonstrate that Rb1 exerts superior therapeutic effects compared to the canonical ferroptosis inhibitor Fer-1 by multi-targeted modulation of ferroptotic pathways. Mechanistically, the protective effect mediated by Rb1 is achieved, at least in part, through direct activation of the NRF2-PPARγ-ACSL4 signaling axis, representing a novel therapeutic approach for AKI intervention.

﻿Our first major finding revealed that ferroptosis accounts for a substantial proportion (45.77%) of cell death in IRI-induced AKI, with predominant localization in renal tubular epithelial cells (Fig. 1). While apoptosis and necrosis have traditionally been considered primary cell death modalities in ischemic injury [31, 32], our findings align with emerging evidence implicating ferroptosis as a major contributor to IRI pathogenesis across multiple organ systems [33]. Given the clinical prevalence of IRI as a leading cause of AKI [25], these observations position ferroptosis inhibition as a promising therapeutic strategy. Although Fer-1 remains the most extensively characterized ferroptosis inhibitor to date, its clinical applicability is limited by chemical instability (ester bond hydrolysis) and unidirectional mechanism (exclusive targeting of lipid peroxidation) [11]. In contrast, our data suggest Rb1 may overcome these limitations through its multimodal action.

Through parallel investigations in IRI-challenged mice and RSL3-stimulated HK-2 cells, we demonstrated Rb1's comprehensive protection against all core aspects of ferroptosis pathology: (1) restoration of the GPX4-glutathione antioxidant system (SLC7A11, GSH, GPX4), (2) normalization of iron homeostasis (TFRC, FTH1, Fe^2^⁺), and (3) suppression of lipid peroxidation (MDA, ROS) (Figs. 3,4). Notably, Rb1 consistently outperformed Fer-1 in modulating these pathways while concomitantly alleviating renal histopathological damage, attenuating inflammatory responses, and improving renal function (Figs. 2, 4). Given that both Fer-1 and Rb1 inhibit RSL3-induced ferroptosis (despite their distinct mechanisms) and that Fer-1 is a well-established positive control, we consider it reasonable to compare Fer-1 and Rb1. As the primary bioactive component of Panax ginseng, a traditional medicine with well-documented clinical safety, Rb1 represents a particularly promising candidate for clinical translation in AKI management [23]. To our knowledge, this study provides the first evidence for Rb1 as a superior ferroptosis inhibitor in AKI pathophysiology.

The therapeutic implications of Rb1 may extend beyond AKI, as compelling evidence links incomplete recovery from AKI to progressive chronic kidney disease (CKD) via persistent tubular injury, oxidative stress and fibroblast activation [34, 35]. Notably, Rb1 has demonstrated efficacy in mitigating oxidative stress and inflammation in CKD models [17, 36], while NADPH oxidase 4 (NOX4) knockout attenuates renal fibrosis by inhibiting fibroblast activation [35]. Considering NOX4's established role in ferroptosis through ROS generation [37], we posit that Rb1's anti-ferroptotic effects may mechanistically contribute to its benefits in early CKD progression.

**Fig. 9:**
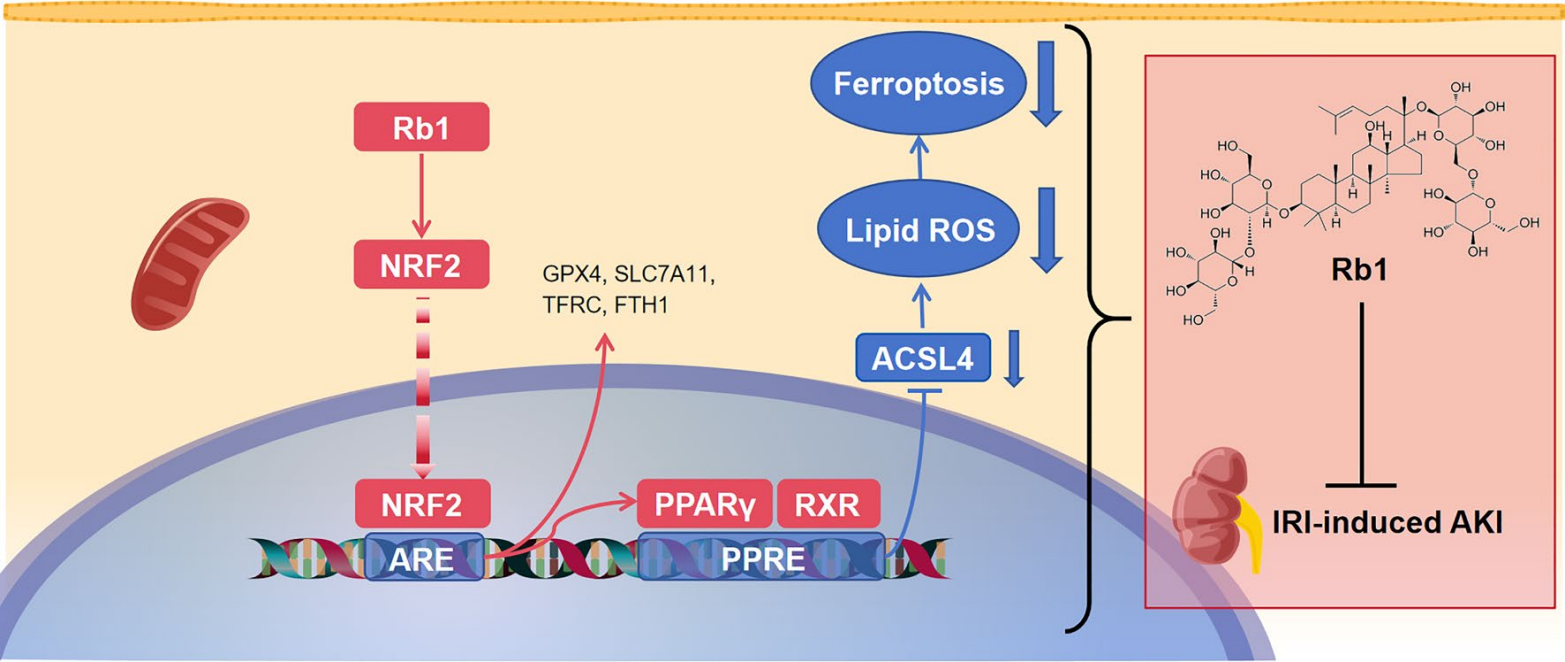
Schematic diagram illustrating the protective role of Rb1 in IRI-induced AKI and its underlying mechanism. GinsenosideRb1 downregulates ACSL4 expression through the NRF2-PPARγ-ACSL4 signaling pathway, thereby inhibiting lipid peroxidation and ferroptosis, which ultimately alleviates IRI-induced AKI (parts of the figure were drawn by using pictures from SciDraw)

Most importantly, our research elucidates a novel molecular mechanism whereby Rb1 exerts its anti-ferroptotic effects through direct activation of the NRF2-PPARγ-ACSL4 signaling cascade (Figs. 5, 6, 7, 8, 9). NRF2, the master regulator of cellular antioxidant responses, mediates cytoprotection by binding to antioxidant response elements (AREs) to initiate gene transcription programs that counteract oxidative damage [38]. While prior investigations attributed Rb1's NRF2 modulation primarily to indirect effects through KEAP1 binding [39, 40], using molecular docking, molecular dynamics simulations and CETSA, our experimental results all showed direct binding between Rb1 and NRF2. We also found that Rb1 promoted both the expression and activation of NRF2, which was demonstrated by the upregulation of its target genes GPX4 and SLC7A11. Notably, although NRF2 must dissociate from KEAP1 for activation, it has also been reported that Rb1 binding to p47^phox^ promotes the p47^phox^/NRF2 complex, which reduces NRF2 degradation and facilitates its nuclear accumulation and activation [41]. Our findings contribute an additional perspective, suggesting that Rb1 can also bind directly to NRF2, potentially enhancing the stability of the p47^phox^/NRF2 complex and subsequently promoting NRF2 nuclear translocation and enhances its expression. However, this remains to be further verified.

Our findings demonstrate that Rb1 orchestrates a dual protective mechanism against ferroptosis through NRF2 activation. As a master regulator of cellular antioxidant responses [38], NRF2 mediates its effects both directly through canonical ferroptosis pathways and indirectly via PPARγ activation. Importantly, PPARγ functions as an ARE-responsive effector downstream of NRF2 [38], regulating gene expression through heterodimerization with retinoid X receptor (RXR) and subsequent binding to peroxisome proliferator response elements (PPREs) [42, 43]. This NRF2-PPARγ signaling axis explains the coordinated upregulation we observed in both transcription factors during Rb1 treatment. Furthermore, results from ChIP assays confirmed NRF2's binding to the PPARγ promoter and its role in activating PPARγ expression. The downstream consequences of this signaling axis are particularly significant in the context of ferroptosis pathogenesis, where ACSL4 has emerged as a critical mediator of lipid peroxidation [8, 38, 44, 45]. Our findings demonstrate Rb1's capacity to markedly suppress ACSL4 expression and subsequent lipid peroxidation in both experimental models. The RNA-seq data reinforcing the connection between PPAR signaling and lipid peroxidation provides additional mechanistic insight. These observations align with published reports that PPARγ activation ameliorates renal pathology in AKI models by reducing lipid accumulation [46]. Moreover, a recent study demonstrated that disruption of the NCOR2-PPARγ interaction upregulates ACSL4 expression, thereby enhancing fatty acid oxidation [47]. These finding provides compelling evidence that PPARγ operates upstream of ACSL4 and negatively regulates its expression, consequently modulating lipid peroxidation levels. Collectively, these observations substantiate our proposed mechanistic pathway through which Rb1 exerts its anti-ferroptotic effects.

We observed that the specific NRF2 antagonist ML385 reversed the Rb1-mediated upregulation of NRF2 and PPARγ, downregulation of ACSL4, and ultimately abolished Rb1's anti-ferroptotic effects. The finding that ML385 not only suppressed NRF2 but also counteracted Rb1's regulation of its downstream targets, PPARγ and ACSL4, validates the hierarchical nature of the NRF2-PPARγ-ACSL4 signaling axis. Therefore, we propose that Rb1 acts, at least in part, by directly activating the NRF2-PPARγ-ACSL4 signaling cascade to inhibit lipid peroxidation, ferroptosis in renal tubular epithelial cells, and the progression of AKI.

In conclusion, our study establishes ferroptosis as a major contributor to tubular injury in IRI-induced AKI and identifies Rb1 as a novel multi-targeted ferroptosis inhibitor with superior efficacy to the current classical Fer-1. Our study demonstrates that Rb1 activates the NRF2-PPARγ-ACSL4 signaling axis, downregulates ACSL4 expression, and thereby suppresses PTECs lipid peroxidation and ferroptosis, ultimately protecting against IRI-induced AKI (Fig. 9). This discovery not only elucidates its novel molecular mechanism, but also provides novel therapeutic targets for AKI intervention. Given its favorable pharmacokinetic profile and established clinical safety, Rb1 represents a promising candidate for clinical translation in ischemia-associated kidney pathologies. Future studies should explore Rb1's potential in other ferroptosis-associated disorders and investigate structure–activity relationships to develop even more potent derivatives.

## Data Availability

The data presented in this study will be shared upon reasonable request. The RNA-Seq data analyzed during this study is available in the NCBI repository (BioProject accession number: PRJNA1244307).

## Abbreviations

Rb1: Ginsenoside Rb1
AKI: Acute kidney injury
IRI: Ischemia–reperfusion injury
PTEC: Proximal tubular epithelial cell
GSH: Glutathione
MDA: Malondialdehyde
ROS: Reactive oxygen species
GPX4: Glutathione peroxidase 4
TFRC: Transferrin receptor
FTH1: Ferritin heavy chain 1
ACSL4: Acyl-CoA synthetase long-chain family member 4
NRF2: Nuclear factor erythroid 2-related factor 2
ARE: Antioxidant response element
PPARγ: Peroxisome proliferator activated receptor γ
RXR: Retinoid X receptor
PPRE: Peroxisome proliferator response element
PUFA-PLs: Polyunsaturated fatty acid-containing phospholipids
